# *Bacillus licheniformis* Anti-TRAP can assemble into two types of dodecameric particles with the same symmetry but inverted orientation of trimers

**DOI:** 10.1016/j.jsb.2010.01.013

**Published:** 2010-04

**Authors:** Mikhail B. Shevtsov, Yanling Chen, Michail N. Isupov, Andrew Leech, Paul Gollnick, Alfred A. Antson

**Affiliations:** a York Structural Biology Laboratory, Department of Chemistry, University of York, Heslington, York YO1 5YW, UK; b Department of Biological Sciences, State University of New York at Buffalo, Buffalo, NY 14260, USA; c School of Biosciences, Henry Wellcome Building for Biocatalysis, University of Exeter, Stocker Road, Exeter EX4 4QD, UK; d Department of Biology, University of York, Heslington, York YO1 5DD, UK

**Keywords:** X-ray crystallography, Transcription attenuation, Tryptophan biosynthesis, *trp* RNA-binding attenuation protein, Anti-TRAP protein, Oligomeric assembly

## Abstract

Anti-TRAP (AT) protein regulates expression of tryptophan biosynthetic genes by binding to the *trp* RNA-binding attenuation protein (TRAP) and preventing its interaction with RNA. *Bacillus subtilis* AT forms trimers that can either interact with TRAP or can further assemble into dodecameric particles. To determine which oligomeric forms are preserved in AT proteins of other *Bacilli* we studied *Bacillus licheniformis* AT which shares 66% sequence identity with the *B. subtilis* protein. We show that in solution *B. licheniformis* AT forms stable trimers. In crystals, depending on pH, such trimers assemble into two different types of dodecameric particles, both having 23 point group symmetry. The dodecamer formed at pH 6.0 has the same conformation as previously observed for *B. subtilis* AT. This dodecamer contains a large internal chamber with the volume of ∼700 Å^3^, which is lined by the side chains of twelve valine residues. The presence of the hydrophobic chamber hints at the possibility that the dodecamer formation could be induced by binding of a ligand. Interestingly, in the dodecamer formed at pH 8.0 all trimers are turned inside out relatively to the form observed at pH 6.0.

## 1 Introduction

In *Bacillus subtilis* tryptophan biosynthesis is regulated by two proteins, the *trp* RNA-binding attenuation protein (TRAP) and Anti-TRAP (AT), which are involved in sensing levels of free tryptophan and uncharged tryptophanyl transfer RNA (tRNA^Trp^), respectively (Gollnick et al., 2005). Tryptophan-activated TRAP halts transcription of the *trp* operon by binding to a leader mRNA segment and facilitating formation of a transcription terminator hairpin (Babitzke and Yanofsky, 1993; Babitzke et al., 1994; Antson et al., 1995). In addition to controlling transcription of the *trp* operon, TRAP inhibits translation initiation of the *trpE* and several other genes that are related to tryptophan metabolism and transport (Gollnick et al., 2005; Merino et al., 1995) by binding to specific sites in their transcripts containing multiple appropriately separated UAG and GAG triplets (Antson et al., 1999; Hopcroft et al., 2002, 2004).

**Fig. 1:**
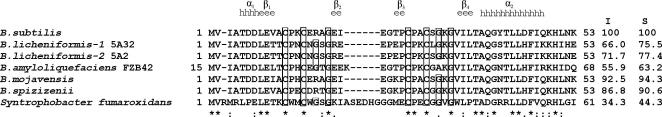
Alignment of AT sequences. AT sequences from seven different Bacilli are shown with Cys and Gly residues of the zinc-binding C–X–X–C–X–G–X–G motif (X designates variable amino acid) highlighted by frames. Secondary structure is designates at the top (h-helix, e-strand).

AT antagonises TRAP action by binding to tryptophan-activated TRAP and preventing its interaction with the leader region of the *trp* mRNA (Valbuzzi and Yanofsky, 2001). *B. subtilis* AT is the product of the *rtpA* gene located within the *at* operon (Valbuzzi et al., 2002). It is expressed in response to elevated levels of uncharged tryptophanyl transfer RNA (tRNA^Trp^), with the expression being controlled by tandem transcriptional and translational mechanisms (Chen and Yanofsky, 2003). AT exhibits sequence homology with the DnaJ protein, which belongs to the type I family of Hsp40 molecular chaperones (Martinez-Yamout et al., 2000). Sequence similarity is largely concentrated within the two zinc-binding cysteine-rich motifs CXXCXGXG which are conserved across AT proteins from different species (Fig. 1). Binding of zinc ion is essential for formation of AT oligomers and for its function (Valbuzzi and Yanofsky, 2002). Earlier we reported the crystal structure of the *B. subtilis* AT at 2.8 Å resolution, showing that this 53-residue protein forms stable trimers that can further assemble into dodecameric particles in which individual trimers are related by 2- and 3-fold symmetry axes (Shevtsov et al., 2005). Individual AT trimers can interact with TRAP (Chen and Gollnick, 2008; Watanabe et al., 2009) but it remained unclear if dodecamers observed in the crystal structure had any biological role.

We reasoned that functionally important oligomeric forms should be conserved among AT proteins from different bacterial species. Here, we report two crystal structures of *Bacillus licheniformis* AT, which shares 66% sequence identity with *B. subtilis* AT (Fig. 1). This protein can form stable complexes with TRAP from *B. subtilis*, *B. licheniformis* and *Bacillus stearothermophilus* (Chen and Gollnick, 2008). We determined structures of the *B. licheniformis* AT at pH 6.0 and 8.0 to resolutions of 2.1 and 2.2 Å, respectively. Remarkably, depending on the pH during crystallisation, AT assembles into two different types of dodecamer, which have the same internal symmetry but inverted orientation of trimers within the oligomer. We show that the dodecamer formed at pH 6.0 is essentially the same as previously observed for *B. subtilis* AT. The conserved nature of this dodecamer hints at its biological significance. The relatively large inter-trimer surface area and the presence of an internal hydrophobic chamber at the centre of the oligomer support such possibility, suggesting that formation of this dodecamer could be induced by binding a ligand.

## 2 Materials and methods

### 2.1 Overexpression, purification and crystallisation

The *B. licheniformis* AT (5A32 strain, Fig. 1) was expressed using a T7 expression system in *Escherichia coli* from pET17b (Novagen) and purified as described (Shevtsov et al., 2004). The protein was dialysed against 1 mM dithiothreitol, 20 mM NaCl and 10 mM triethanolamine pH 8.0 using a mini dialysis units (Pierce) with molecular weight cut-off of 7 kDa and concentrated to 20 mg/ml using ultrafiltration column (Vivascience). Due to the low extinction coefficient of the protein, which lacks tryptophan and tyrosine residues, its concentration was estimated using the Bradford reagent (Pierce) with the bovine serum albumin as a standard (Bradford, 1976).

Crystallisation searches were performed using sparse-matrix screens (Jancarik and Kim, 1991) set up in 96-well Greiner microplates (Greiner Bio-One) using the Mosquito crystallization robot (TTP LabTech). Each droplet was formed by mixing equal volumes (150 nl) of protein solution and reservoir. Crystals with two different morphologies grew at pH 6.0 and 8.0. Crystallisation was optimized in hanging drops, prepared by mixing 1 μl of protein solution with 1 μl of reservoir which contained 1 ml of solution. The best crystals at pH 6.0 were obtained using 0.1 M Bis–Tris and 23–27% of poly(ethylene glycol) 3350 in the reservoir. The best crystals at pH 8.0 were grown using 0.1 M Tris–HCl, 0.2 M MgCl_2_ and 20–25% poly(ethylene glycol) 3350 under increased gradient (and hence increased diffusion rate) achieved by addition of 0.5 M NaCl into reservoir before sealing the crystallisation container.

### 2.2 X-ray data collection, structure determination and analysis

Crystals were flash-frozen using cryo solutions with matching concentrations of all the ingredients present in the crystallisation drop, but with 3% higher concentration of precipitating agent (PEG) and with addition of 16% (v/v) glycerol. X-ray data were collected using synchrotron radiation, Table 1. The data were processed using DENZO/SCALEPACK (Otwinowski and Minor, 1997) and MOSFLM/SCALA (Evans, 1997; Leslie, 1993) in the case of low- and high-pH forms, respectively.

**Supplementary Fig. S1:**
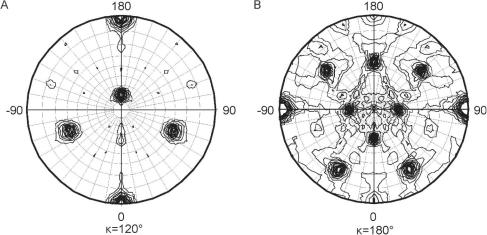
Stereographic projection of the self-rotation function for the high-pH crystal form of *B. licheniformis* AT. The resolution was 10.0–3.0 Å and the radius of integration was 30 Å. Contours start at zero and increase in 0.6*σ* intervals. Crystallographic *a* axis is along *ϕ* = 0° *ψ = *90° and the *c*∗ axis is perpendicular to the plane of the figure. The *ϕ* angle varies from 0° to ±180° around the plot while the *ψ* angle varies from 0° at the centre to 90° at the edge. (A) Section with *κ *= 120°, (B) Section with *κ *= 180°.

Structures of both crystallographic forms were solved using MOLREP (Vagin and Teplyakov, 1997) using the *B. subtilis* AT trimer (Shevtsov et al., 2005) as a search model. The molecular replacement solution for the low-pH form (H3 space group) was found using automatic mode. Solution of the high-pH form (P2_1_ space group) proved to be more difficult. The self-rotation function calculated for this crystal form indicated that the *B. licheniformis* AT molecule is a dodecamer with 23 point group symmetry (Supplementary Fig. S1). The native Patterson synthesis calculated for the 14–4 Å resolution shell contained peaks at (0.5, 0.129, 0.0) and (0.5, 0.0, 0.5) with the height of 0.4 of the origin peak and peak at (0.0, 0.130, 0.5) with the height of 0.16 of the origin. Such peaks together with analysis of the solvent content (Matthews, 1968) suggested the presence of up to four dodecamers (4 × 12-subunits) in the asymmetric unit, which are related by pseudotranslation. Despite the relatively high 66% sequence identity, no solution was found for MR searches with monomers, trimers or dodecamers of *B. subtilis* AT. Therefore, we attempted to determine the structure using the NCS-constrained procedure (Isupov and Lebedev, 2008). The *B. subtilis* AT trimer was positioned so that its molecular threefold axis was parallel to one of the NCS threefold axes. The trimer was rotated around the axis with an increment of 2° in the range 0–120° and a translation search was carried out for each orientation. The correct solution had correlation of 9.4% while the best solution for other orientations had correlations of 6.2% or less. The trimer was fixed in the correct orientation and the search repeated for molecules related by pseudotranslation and for trimers aligned with other NCS threefold axis. This approach led to correct positioning of 16 trimers, which form 4 dodecamers related by pseudotranslation. Although these dodecamers had the same point group symmetry as the original *B. subtilis* model, the trimers were organised in a completely different way, explaining why it was not possible to solve the structure using standard approaches.

Model building was performed using X-AUTOFIT (Oldfield, 1994) implemented in QUANTA (Accelrys Inc.). Structures were refined by REFMAC (Murshudov et al., 1997) using the TLS option (Winn et al., 2000) at the end of the refinement process. Refinement of the high-pH form stalled at *R/R*_free_ = 33/43% in a false minimum due to poor convergence caused by the pseudotranslation. At this stage further improvement was obtained by using the best defined dodecamer (out of four) for further molecular replacement followed by refinement. NCS restraints were imposed during early stages of refinement, with several final cycles performed without such restraints. Distances between zinc atoms and the coordinating sulphur atoms of cysteines were constrained to 2.34 Å (Harding, 2006).

Difference electron density maps of the high-pH form contained 16 significant peaks, one per trimer, coordinated by the main-chain carboxyl groups of three C-terminal residues (Glu53). These peaks appeared at a level of ∼4.5*σ* in well-ordered parts of the structure. The composition of the crystallisation solution suggested that these peaks correspond to Mg^2+^ ions, in agreement with coordinating distances of 2.2–2.5 Å normally observed for Mg^2+^ ions (Harding, 2006). Analysis of anomalous difference maps did not reveal any significant peaks at the positions of metal ions, as expected at 0.87 Å wavelength for Mg^2+^; the refinement of magnesium ions present in well-ordered regions of oligomers resulted in temperature factors that were in good correspondence with the temperature factors of coordinating oxygen atoms, further supporting assignment of peaks to Mg^2+^ ions. Electron density of several magnesium-coordinated sites indicated partial disorder. Coordinating distances at these sites were constrained to 2.18 Å during the refinement.

Dodecameric assemblies observed in crystal structures were analysed using the PISA web server (Krissinel and Hendrick, 2007). All figures were generated using PYMOL (DeLano Scientific, 2002).

### 2.3 Characterisation of AT assemblies by gel filtration

The oligomeric state of *B. licheniformis* and *B. subtilis* AT in solution was analysed using a Superose 12 HR 10/30 column (Amersham Bioscience) and the ÄKTApurifier™ HPLC (GE Healthcare). The running buffer contained 20 mM Bis–Tris propane (pH 6.0 and 8.0) and high or low salt concentrations (50 and 250 mM of potassium chloride). Due to the low extinction coefficient of both *B. subtilis* and *B. licheniformis* AT at 280 nm, the elution was monitored at 215 nm, in addition to the standard 260 and 280 nm. The concentrations of loaded samples of *B. subtilis* and *B. licheniformis* AT were 4 and 8 mg/ml, respectively.

### 2.4 Analytical ultracentrifugation

For analytical ultracentrifugation (AUC) experiments samples of *B. licheniformis* AT were prepared using conditions similar to those used during crystallisation: (1) 0.1 M Bis–Tris pH 6.0, 20 mM NaCl; (2) 0.1 M Tris pH 8.5, 20 mM NaCl and (3) 0.1 M Tris pH 8.0, 20 mM NaCl, 20 mM MgCl_2_. The protein concentration in all samples was 3 mg/ml or ∼0.17 mM in [AT]_3_ units (estimated using Bradford reagent). Sedimentation velocity experiments were conducted at 20 °C on a Beckman Optima XL/I analytical ultracentrifuge, using Beckman cells with 12 mm path length double sector charcoal-filled Epon centrepieces and sapphire windows, in an AN-50Ti rotor (four cells plus the counterbalance). Four hundred and twenty microliters of reference buffer and a slightly smaller volume (416 μl) of the sample were loaded into the cells. Prior to the run absorbance scans were taken at 3000 rpm to check loading concentrations, and ensure that the cell contents were uniformly distributed. The speed of rotation was then increased to 4000 rpm, and interference scans (for all cells) and absorbance scans (280 nm) were taken at approximately 3 min intervals until sedimentation was complete (until the plateau region had disappeared). The duration of sedimentation experiment was 6.5 h. Due to the low extinction coefficient of the protein, absorbance scans were performed at 245 nm and data were collected using the interference optical system. Partial specific volumes, buffer densities and viscosities were estimated using the program SEDNTERP (Laue et al., 1992). Data were analysed by SEDFIT (Schuck, 2000) using the c(s) model (continuous distribution of sedimentation coefficients).

### 2.5 PDB Accession codes

Coordinates and structure factors have been deposited with the Protein Data Bank under accession codes 3LCZ and 3LD0 for the low-pH and high-pH crystal forms, respectively.

## 3 Results

**Fig. 2:**
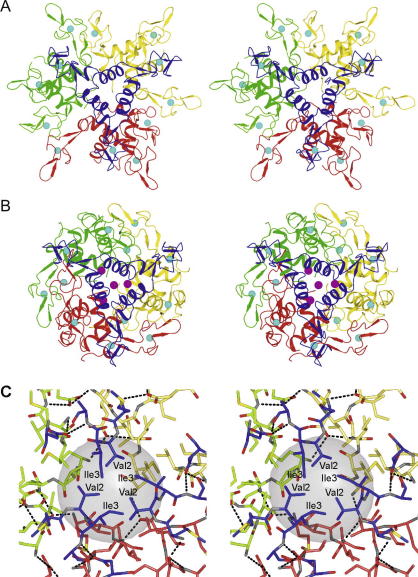
Two different types of AT dodecamer. Stereo diagrams are shown with individual trimers coloured differently, zinc atoms shown by cyan spheres and magnesium ions by magenta spheres. (A) Ribbon diagram of the low-pH form. (B) Ribbon diagram of the high-pH form. (C) Central chamber in the low-pH dodecamer shown in the same orientation as in (A). Stereo diagram is shown with Met1, Val2, Ile3, Ala4 and Asp7 of all 12-subunits shown as sticks. The central Val2 and Ile3 residues are labelled for the trimer coloured in blue. The semitransparent sphere with the diameter of 11 Å is positioned at the center of the internal chamber. Hydrogen bonds formed by pairs of residues Met1–Asp7 (from different trimers) and Met1–Ala4 (from the same trimer) are represented by dashed lines.

Structures of *B. licheniformis* AT determined at the two different pH values of 6.0 and 8.0 (Fig. 2A and B), are further referred to as the “low-pH” and “high-pH” crystal forms. The low-pH crystals belong to the space group H3 with four AT monomers per asymmetric unit whereas the high-pH crystals belong to the space group P2_1_ and contain 48 AT monomers in the asymmetric part. The low- and high-pH structures were refined at resolutions of 2.06 Å (*R/R*_free_ = 21.6/28.6%) and 2.2 Å (*R/R*_free_ = 19.5/25.9%), respectively, Table 1.

### 3.1 Crystal structure at pH 6.0

Three of the four monomers in the asymmetric unit constitute a trimer. Packing of the trimer and the additional AT subunit around the crystallographic 3-fold axis generates four AT trimers that form a dodecameric particle (Fig. 2A). The architecture of this dodecamer is identical to the one observed for *B. subtilis* AT at pH 6.5 (Shevtsov et al., 2005). The r.m.s. difference between the main-chain atoms of the core parts of the two dodecamers (excluding the zinc-binding residues 9–36 and C-terminal residues 50–53) varies from 0.7 to 1.5 Å, depending on their orientation during superposition. The inclusion of the zinc-binding domains and the C-terminal residues results in significantly larger r.m.s. difference of 2.0–8.0 Å. This is not surprising as zinc-binding domains have a rather flexible nature as indicated by their significant conformational variation and high temperature factors in the structure of *B. subtilis* AT (Shevtsov et al., 2005).

**Fig. 3:**
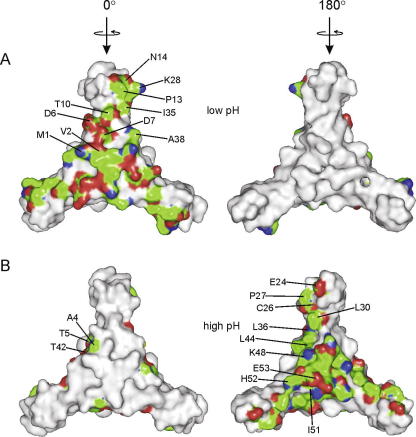
Trimer–trimer interacting surfaces in the two dodecameric forms of the *B. licheniformis* AT. AT trimer is represented by a molecular surface. Residues involved in the formation of dodecamers are labelled and coloured according to the type of interaction between the trimers, with residues forming van der Waals interactions shown in green and residues forming hydrogen bonds shown in red (acceptors) and blue (donors). The threshold distance for determining the contacts was 3.5 Å. The low-pH form is shown in (A) and the high-pH form is shown in (B). (For interpretation of the references to color in this figure legend, the reader is referred to the web version of this paper.)

As with *B. subtilis* AT, trimer–trimer association within the *B. licheniformis* dodecamer is maintained mainly by hydrophobic interactions in two regions. The first is a hydrophobic area in the centre of the molecule (Fig. 2C), lined by residues 1–3 from all 12 AT monomers. The second region of stabilising interactions includes van der Waals contacts between pairs of adjacent zinc-binding domains formed along the 2-fold molecular axes by residues Pro13 and Ile35 (Fig. 3A). In addition, there are inter-trimer hydrogen bonds formed between two pairs of residues, Met1–Asp7 and Asn14–Asn14.

An interesting feature of the low-pH dodecamer is the relatively large internal chamber inside the multimeric assembly, which is centred at the intersection of symmetry axes and is isolated from the solvent (Fig. 2C). There are no peaks in the electron density maps inside the chamber suggesting that it is filled by disordered crystallisation buffer components or solvent. Its inner surface is lined by the side chains of twelve Val2 residues, this amino acid is strictly conserved in AT proteins (Fig. 1). The chamber could be fitted with a sphere of ∼11 Å in diameter, potentially permitting the accommodation of a relatively large ligand (Fig. 2C).

### 3.2 Crystal structure at pH 8.0

The asymmetric unit of the high-pH form contains four AT dodecamers (48 subunits), all with identical architecture. The high-pH dodecamer (Fig. 2B) has the same 23 point group symmetry as the low-pH dodecamer, but the orientation of trimers within the oligomer is inverted so that residues buried in the low-pH oligomer become exposed in the high-pH oligomer and vice versa (Fig. 3). In contrast to the low-pH form, the zinc-binding domains of the high-pH dodecamer participate in trimer–trimer interactions while their C-terminal helical regions are buried inside the dodecamer. Interestingly, the conformation of individual trimers in both dodecamers is essentially the same, with a typical main-chain atoms r.m.s. difference of 1.3 Å (0.4 Å for core segments excluding the zinc-binding domains). In contrast to the low-pH form, the high-pH dodecamer does not contain any significant internal cavities and has a more compact structure. Trimer–trimer contacts include a number of hydrogen bonds, formed between pairs of residues Thr5–Glu24, Glu24–Thr42, Lys48–His52, Ile51–Glu53 and Cys26–Ser41 (Fig. 3B). There are also hydrophobic interactions between pairs of residues Leu30–Leu36, Leu30–Leu44.

The high-pH form contains Mg^2+^ ions, coordinated by the C-terminal carboxyl groups of three subunits. From the crystal structure alone it is not clear whether the formation of the 12-mer is induced by these ions although it is likely that they stabilise the conformation of His52, which is located in a disallowed region of the Ramachandran plot.

Zinc-binding segments (residues 9–36) of the high-pH dodecamer are more flexible than the core area of the trimer despite their involvement in trimer–trimer interactions. This is evidenced by the temperature factors of their main-chain atoms, which are increased by 7–30 Å^2^, depending on a particular subunit. At the same time, the zinc-binding segments are significantly less flexible than in the low-pH form where they are more exposed.

### 3.3 Analysis of oligomeric states *in silico* and *in vitro*

A monomer of *B. licheniformis* AT has a surface area of ∼4300 Å^2^, of which ∼1300 Å^2^ are buried in intersubunit contacts within the trimer. The total surface area of each trimer is ∼9000 Å^2^, of which ∼1700 Å^2^ (∼19% of the total) or 2800 Å^2^ (∼31% of the total) are contributed to the formation of the low-pH and high-pH dodecamers, respectively. The analysis of the low-pH dodecameric assemblies of *B. subtilis* and *B. licheniformis* AT using PISA (Krissinel and Hendrick, 2007) resulted in the Δ*G*_diss_ values of 23.4 and 20.7 kcal/mol, respectively. Similar analysis of the high-pH dodecamer of *B. licheniformis* AT resulted in the Δ*G*_diss_ value of 38.2 kcal/mol. This analysis and the very significant surface areas buried in trimer–trimer interactions indicated that dodecameric assemblies observed in crystal structures might be energetically stable and biologically relevant.

**Supplementary Fig. S2:**
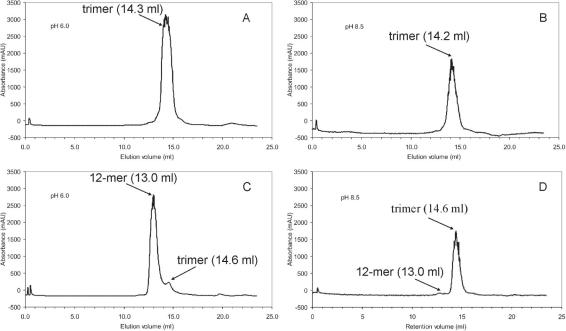
Gel-filtration profiles of *B. licheniformis* and *B. subtilis* AT. Profiles for *B. licheniformis* AT are shown for pH 6.0 (A) and pH 8.5 (B). Profiles for *B. subtilis* AT are shown for pH 6.0 (C) and pH 8.5 (D). Profiles were recorded at 215 nm.

**Supplementary Fig. S3:**
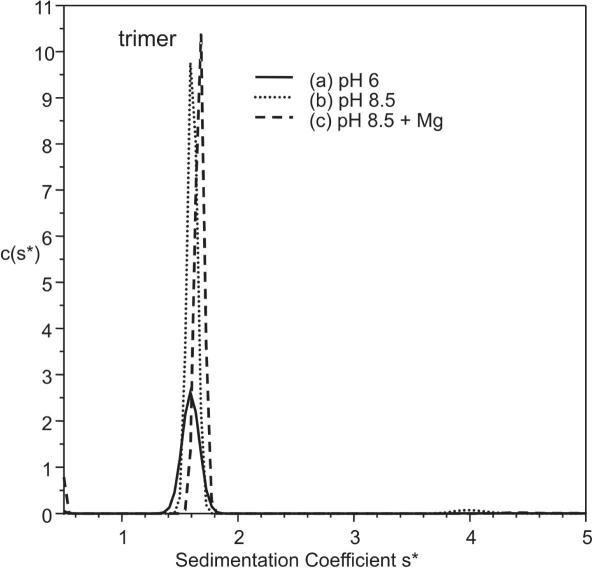
Sedimentation velocity analysis of AT. The sedimentation coefficient of 1.6 corresponds to the AT trimer (∼17 kDa).

We characterised both *B. subtilis* and *B. licheniformis* AT in solution at low (6.0–6.5) and high (8.0–8.5) pH. The influence of low (50 mM) and high (250 mM) concentrations of potassium chloride on oligomeric states of *B. subtilis* and *B. licheniformis* AT was also tested. Gel-filtration profiles showed that *B. subtilis* AT exists in equilibrium between trimeric and dodecameric species (Supplementary Fig. S2). This equilibrium did not depend on salt concentration (data not shown), but depended on the pH of the solution, shifting towards formation of trimers at pH 8.0 or above. Similar experiments with *B. licheniformis* AT revealed only trimeric species at all conditions tested. In agreement, AUC experiments with *B. licheniformis* AT (Supplementary Fig. S3) showed that at both pH 6.0 and 8.0 the protein was present as a 1.6 S form, corresponding to AT trimers; addition of Mg^2+^ ions did not influence the sedimentation profile. There was a trace of larger oligomers (<1%) present at pH 8.5.

## 4 Discussion

Structural data reported here show that *B. licheniformis* AT forms stable trimers, which have essentially the same conformation as trimers of *B. subtilis* AT. The trimer is the functional unit of AT that interacts with TRAP (Chen and Gollnick, 2008; Watanabe et al., 2009). Consistent with this, such trimers were observed in solution during gel-filtration and AUC experiments. We also show that at high concentrations present during crystallisation, *B. licheniformis* AT trimers can assemble into two different 12-subunit particles. We attempted to identify why the dodecamers have different arrangements by analysing protonation states of individual residues that might select one form over the other. As described in Section 3, interactions between individual trimers of the low-pH dodecamer include a salt bridge between the amino-terminal group of Met1 and the side chain carboxyl of Asp7 (Fig. 3A). The calculated p*K*_a_ value for the amino group of Met1 is 7.1 (Gordon et al., 2005). This indicates that the salt bridge formation is favoured in case of the low-pH crystal form (pH 6.0) as the amino group of Met1 is likely to be protonated. Identical Met1–Asp7 salt bridge interaction was observed in the structure of *B. subtilis* AT at pH 6.5 (Shevtsov et al., 2005). At pH 8.0 the salt bridge interaction is less likely as the majority of the amino-terminal groups are expected to be unprotonated, allowing different packing of individual trimers.

The question arises if any of the two dodecameric forms are biologically significant and whether these could be involved in regulation? The conformation of the 12-subunit assembly of *B. licheniformis* AT observed at pH 6.0 is essentially the same as previously seen for *B. subtilis* AT (Shevtsov et al., 2005), in spite of the significant variation in the sequence (66% identity between the two proteins). Surfaces involved in interaction with TRAP are partially buried, thus the oligomer formation could prevent interaction with TRAP. Formation of these dodecameric assemblies is consistent with gel-filtration experiments presented here showing that *B. subtilis* AT exists in equilibrium between trimeric and dodecameric forms. On the other hand, the AT concentration required for formation of these oligomers is ∼10 times higher (Snyder et al., 2004) than the estimated cellular concentration of AT (Yang and Yanofsky, 2005). Moreover, in the case of *B. licheniformis* AT, the dodecameric assemblies were not observed by gel filtration and AUC, thus questioning the biological relevance of the 12-subunit particles. In contrast to these observations, the analysis of the low-pH dodecameric assemblies by PISA (Krissinel and Hendrick, 2007) indicates that these might be energetically stable and thus biologically relevant. In accordance, the relative percentage of solvent accessible area contributed to the formation of dodecameric assemblies is very significant (Jones and Thornton, 1996). Finally the presence of the large internal chamber lined up by hydrophobic residues suggests that formation of 12-subunit particles could be induced by yet unknown ligands that may stabilise the assembly.

The *in silico* analysis of the high-pH dodecamer of *B. licheniformis* AT also indicated that it could be relatively stable; this is supported by the significant surface areas buried at trimer–trimer interfaces. To investigate if the high-pH dodecamer can be formed by *B. subtilis* AT, we generated its model by superimposing trimers of *B. subtilis* AT with corresponding trimers in the *B. licheniformis* dodecamer. Analysis of trimer–trimer interactions within the resulting *B. subtilis* dodecamer did not reveal any significant steric clashes, suggesting that it can form the same type of dodecamer.

## 5 Conclusions

The data presented here show that *B. licheniformis* AT forms stable trimers, which have essentially the same conformation as *B. subtilis* AT. We also show that *in vitro* *B. licheniformis* AT exists as a trimer, the oligomeric form that interacts with TRAP (Chen and Gollnick, 2008; Watanabe et al., 2009). At high concentrations used during crystallisation AT forms two different types of dodecameric assemblies. These assemblies bury substantial surface areas, with PISA analysis hinting at their biological significance. The oligomer observed at pH 6.0 is conserved in both *B. subtilis* and *B. licheniformis* AT and contains large internal chamber lined up by hydrophobic residues (Fig. 2C). It is possible that this oligomeric assembly is stabilised by yet unknown cellular factors and could thus be involved in regulatory networks.

## Figures and Tables

**Table 1:** Data collection and structure determination statistics.

*Data collection*		
Crystal form (pH)	6.0	8.0
Beam line	BM14, ESRF, Grenoble	9.6, SRS, Daresbury
Space group	H3	P21
Wavelength (Å)	1.28	0.87
Unit-cell parameters (Å, °)	*a = *108.1, *c = *49.4	*a = *118.5, *b = *99.9, *c = *123.2 *β = *117.6
Resolution (Å)^a^	54.0–2.06 (2.13–2.06)	38.2–2.2 (2.32–2.20)
No. of unique reflections	13005 (1265)	128724 (18742)
Completeness (%)	97.8 (95.1)	99.8 (97.0)
*R_merge_* (%)^b^	6.2 (35.5)	7.4 (28)
Average *I/σ(I)*	19.2 (4.1)	11.9 (4.4)
Multiplicity	5.2 (4.1)	3.9 (3.9)

*Model building and refinement*		
Resolution range (Å)	28.8–2.06	29.3–2.2
Total no. of reflections used in refinement	12301	127405
No. of reflections excluded from refinement	647	1300
Protein atoms	1607	19128
Water molecules	113	1336
Zinc/magnesium atoms	4/none	48/16
*R*/*R_free_* (%)^c^	21.6/28.6	19.5/25.9
Wilson *B*-factor after scaling (Å^2^)	46.7	28.5
Average *B* (total, Å^2^)	62.0	25.8
r.m.s. deviation from ideal values^d^		
Bond lengths (Å)	0.012 (0.022)	0.008 (0.022)
Bond angles (°)	1.8 (2.0)	1.2 (2.0)

a Values in parentheses are for the highest resolution shell.

b *R_merge_* = ∑*_hkl_*∑*_i_*|*I_i_*(*h*) − <*I*(*h*) *>* |/∑*_hkl_*∑*_i_I_i_*(*h*), where *I*(*h*) is intensity of reflection *h*, <*I*(*h*)*>* is average value of intensity, the sum ∑*_hkl_* is over all measured reflections and the sum ∑_i_ is over *i* measurements of a reflection.

c Crystallographic *R = *∑*_hkl_*||*F_obs_* − *F_calc_*||/∑*_hkl_*|*F_obs_|*, *R_free_* was calculated using a randomly chosen set of reflections that were excluded from the refinement.

d Target values given in parentheses.
